# Development of Screen-Printed Texture-Barrier Paste for Single-Side Texturization of Interdigitated Back-Contact Silicon Solar Cell Applications

**DOI:** 10.3390/ma6104565

**Published:** 2013-10-17

**Authors:** Yu-Shun Chiu, Chin-Lung Cheng, Thou-Jen Whang, Chi-Cheng Chen

**Affiliations:** 1 Department of Chemistry, National Cheng Kung University, Tainan 70101, Taiwan; E-Mail: L38991081@mail.ncku.edu.tw; 2 Department of Electro-Optical Engineering, National Formosa University, Huwei, Yunlin 63201, Taiwan; 3 Institute of Mechanical and Electro-Mechanical Engineering, National Formosa University, Huwei, Yunlin 63201, Taiwan; E-Mail: d90542@yahoo.com.tw

**Keywords:** screen-printed texture-barrier (SPTB), interdigitated back-contact (IBC), photovoltaic silicon solar cells, paste

## Abstract

Continuous cost reduction of silicon-based solar cells is needed to lower the process time and increase efficiency. To achieve lower costs, screen-printed texture-barrier (SPTB) paste was first developed for single-side texturization (ST) of the interdigitated back-contact (IBC) for silicon-based solar cell applications. The SPTB paste was screen-printed on silicon substrates. The SPTB paste was synthesized from intermixed silicate glass (75 wt %), a resin binder (ethyl cellulose ethoce: 20 wt %), and a dispersing agent (fatty acid: 5 wt %). The silicate glass is a necessity for contact formation during firing. A resin binder and a dispersing agent determine the rheology of the SPTB paste. In this work, by modulating various parameters, including post SPTB firing, alkali texturing, and removal of the SPTB, the ST of IBC silicon solar cells was achieved. Since the advantages of the SPTB paste include low toxicity and prompt formation of the texture-barrier, SPTB is potentially suited for simple fabrication at low-cost for solar cell applications. The cost of the SPTB is around $100/kg which is lower than the SiH_4_/NH_3_ gas ambient used in plasma-enhanced chemical vapor deposition (PECVD). Thus, the expensive Si_3_N_4_ film deposited by PECVD using SiH_4_ and NH_3_ gas ambient for silicon solar cells can be replaced by this SPTB.

## 1. Introduction

Screen-printed mono- and multi-crystalline silicon solar cells have been widely used for industrial solar cells [1,2,3,4,5,6,7]. However, both the front contact and rear side of the screen-printed silicon solar cells (SPSSCs) have been the major obstacle for conversion efficiency (CE) enhancements. This is due to optical loss of the top-contact coverage shading and poor surface recombination. Since maximum CE of the screen-printed mono-crystalline silicon solar cells is around 19.5%, the improvement of SPSSC efficiency is highly desirable. Thus, a CE of 24.2% for interdigitated back-contact (IBC) silicon solar cells has attracted great attention [8,9,10]. Compared with conventional solar cells with front side metal grids, IBC solar cells with interdigitated contact of opposite polarity on the back surface of the cells have various advantages, including improved photo-generation, reduced grid series resistance, improved blue photo-response, and simplified module assembly [11]. In recent years, the IBC solar cells have cells made with aluminum-alloyed emitters using screen-printed technology [12,13]. Moreover, the random pyramids with height of around 2–10 μm in alkali texturing solution were overly larger for the rear side metallization of the IBC solar cells. Therefore, single-side texturization (ST) in IBC solar cells with a flattened back side is preferable. Previous literature reported that the ST on the front surface of the IBC solar cell was achieved by covering a SiN*_x_* protection layer on the rear side of the silicon substrate [13,14]. The high costs of a SiN*_x_* texture-barrier as deposited by plasma-enhanced chemical vapor deposition (PECVD) are here identified. The cost of the screen-printed texture-barrier (SPTB) is around $100/kg which is lower than the SiH_4_/NH_3_ gas ambient used in PECVD. Following this, our study demonstrates a SPTB paste that was fabricated simply at low-cost using screen-printed technology.

## 2. Experimental Section

To develop the SPTB paste for ST applications of the IBC silicon solar cells, all square samples with 4 cm^2^ were cut from (100)-oriented p-type monocrystalline silicon wafer with ρ = 0.5 − 3 Ω cm. The sheet resistance of the p-type Si(100) substrate was measured to be around 130 Ω/sq. Prior to the SPTB process, all wafers were cleaned with a wet process. Wet processes, including an H_2_SO_4_/H_2_O_2_ mixture in a ratio of 4:1 (volume) at 120 °C, an NH_4_OH/H_2_O_2_/H_2_O mixture in a ratio of 1:4:20 (volume) at 75 °C and an HCl/H_2_O_2_/H_2_O mixture in a ratio of 1:1:6 (volume) at 75 °C, were used to remove residues and contaminants. The dipping time in all chemical processes was 10 min. Diluted HF (DHF) with HF and H_2_O in a ratio of 1:100 (volume) was used to remove native oxide. Following chemical processes, all wafers were rinsed with deionized water for 10 min. Then, the prepared SPTB paste was screen-printed onto the cleaned wafer. The process of making SPTB consists of mixing silicon glass powder (75 wt %), organic resin binder (ethyl cellulose ethoce: 20 wt %), and dispersing agent (fatty acid: 5 wt %) in an agitation tank. The ready-mixed materials are dispersed in a roll mill to finally form the SPTB paste. The silicate glass in the SPTB paste is a necessity for contact formation during firing. A resin binder and a dispersing agent determine the rheology of the SPTB paste. The viscosity of the SPTB paste can be tuned by the ratio of the silicate glass, the resin binder, and the dispersing agent. Then, the stacked SPTB paste/p-type Si(100) substrates were fired at 840 °C for 10, 15, 20, 30, 40, 60, 80 and 100 min, respectively. Furthermore, the SPTB paste/p-type Si(100) substrates were textured in a mixture solution of 1.7% potassium hydroxide (KOH) for the ST process. The temperature and the texturing time of the mixture solution were maintained at 83 °C for 15, 20, 25 and 30 min, respectively. After the ST process, the SPTB pastes protected on p-type Si(100) substrates were removed by the mixed HCl/H_2_O_2_ solution at 10, 30 and 60 min, respectively. The process used an HCl/H_2_O_2_/H_2_O mixture in a ratio of 1:1:4 (volume) at 75 °C to remove the SPTB protected layer. Finally, all surface morphologies and cross-section images were detected by a scanning electron microscopy (SEM) (Hitachi, S3000, Tokyo, Japan). All sheet resistances of the surfaces were evaluated by four-point probe current-voltage meters. Since the SPTB paste is similar to the Al paste used in mono-crystalline silicon solar cells, a large size wafer such as 156 × 156 mm^2^ can be screen-printed by design of the large screen.

## 3. Results and Discussion

**Figure 1:**
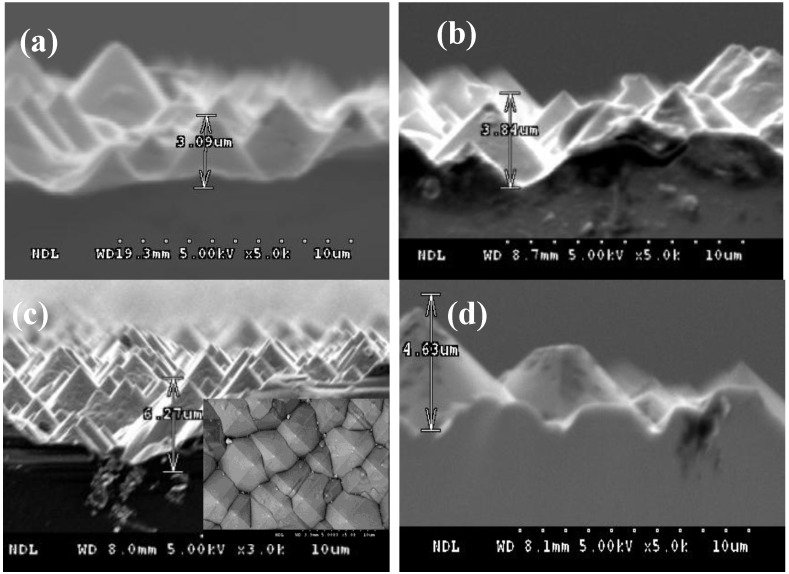
Cross-section images of the p-type Si(100) substrate after texturing at (**a**) 15, (**b**) 20, (**c**) 25 and (**d**) 30 min, respectively, were detected by scanning electron microscopy (SEM). The surface morphologies of the p-type Si(100) with the texturing time of 25 min are shown in the inset of (c).

Firstly, to confirm the quality of the texturization, the p-type Si(100) substrates were textured in a mixed solution of 1.7% KOH. The temperature and texturing time of the texturing solution were maintained at 83 °C for 15, 20, 25 and 30 min, respectively. The cross-section images detected by SEM for (a) 15, (b) 20, (c) 25 and (d) 30 min, respectively, are shown in Figure 1.

The height of the pyramid was measured to be around 1–7 μm. Since pyramids are formed by anisotropical KOH etching on a single crystalline silicon (111) surface, the height of the pyramid was increased by increasing texturing time. Compared with the sample textured at 25 min, a lower height of the pyramid was obtained for samples textured at 15 and 20 min. On the other hand, some top conoids of the pyramid were etched for the sample textured at 30 min, as shown in Figure 1c. The reflection could be increased by the flattened top surface of the pyramid. Thus, according to the surface morphologies and the cross-section image detected by SEM as shown in Figure 1, a suitable texturing time of 25 min would be expected in this work. Therefore, the ST process of the IBC silicon solar cells is achieved at 83 °C for 25 min.

**Figure 2:**
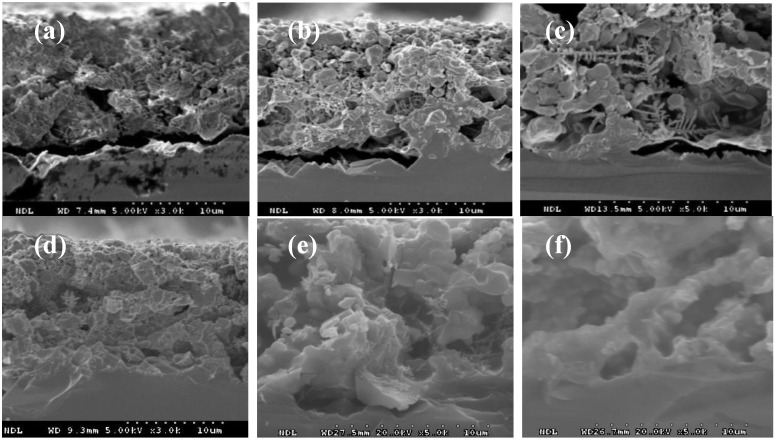
Cross-section images of screen-printed texture-barrier (SPTB) layer/p-type Si(100) substrates fired at 840 °C for (**a**) 10, (**b**) 20, (**c**) 40, (**d**) 60, (**e**) 80, and (**f**) 100 min, respectively, and textured in a 1.7% KOH solution for 25 min.

**Figure 3:**
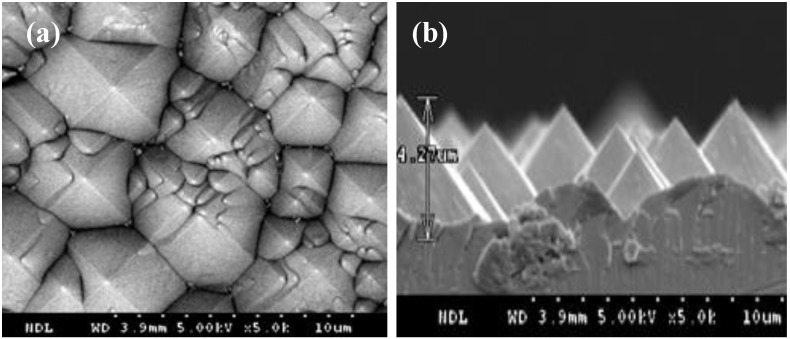
The SPTB layer/p-type Si(100) substrates were textured in 1.7% KOH solution for 25 min. (**a**) the surface morphology and (**b**) the cross-section image of the p-type Si(100) substrate without a SPTB layer was detected by SEM.

To demonstrate the ST of the IBC silicon solar cell, all SPTB layer/p-type Si(100) substrates with firing at 10, 20, 40, 60, 80, and 100 min, respectively, were textured in a 1.7% KOH solution for 25 min. Figure 2 shows the cross-section images of all SPTB layer/p*-*type Si(100) substrates. Figure 3 shows the surface morphologies and the cross-section images of the p-type Si(100) substrates without a SPTB layer side. The height of the pyramid was measured to be around 4∓7 μm. It can be clearly demonstrated that the SPTB layer/p-type Si(100) substrates fired below 60 min cannot be adopted for ST due to the KOH solution that passes through the voids in the SPTB layer (Figure 2a–c). On the contrary, there is no texturization between the SPTB layer/p-type Si(100) substrates interface for the SPTB layer fired above 60 min (Figure 2d,f). The adhesion between the SPTB layer/p-type Si(100) substrate interface increases on increasing the firing time. Therefore, the SPTB layer/p-type Si(100) substrate fired at 840 °C for 60 min are suitable for the preparation of the ST.

**Figure 4:**
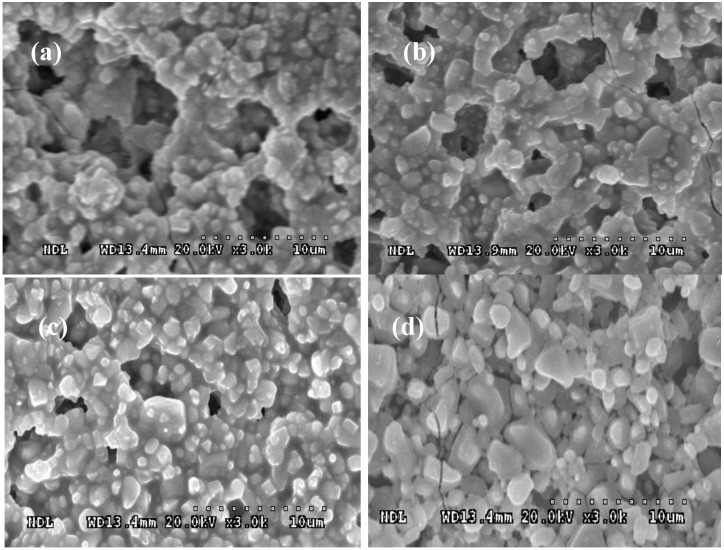
Surface morphologies of screen-printed SPTB on p-type Si(100) substrates fired at 840 °C for (**a**) 10, (**b**) 15, (**c**) 30, and (**d**) 60 min, respectively.

To investigate the surface morphologies of the SPTBs deposited on the p-type Si(100) substrates with various firing times, the stacked SPTB/p-type Si(100) substrate structures were fired at 840 °C for (a) 10, (b) 15, (c) 30 and (d) 60 min, respectively, as shown in Figure 4. The results show that many voids in the SPTB layers were detected for the 10 min sample. The voids in the SPTB layers decreased with increasing firing time of the SPTB layer as shown in Figure 4d. This is due to the flow of the SPTB paste determined by high-temperature firing. When alkali texturing is presented, the KOH solution passes through the voids in the SPTB layer and then etches the silicon surface. On the other hand, the SPTB layer with long time firing is difficult to remove after single-side texturization. Fewer voids result in shorter etching times of the silicon surface, thus introducing a trade-off between maintaining fewer voids and achieving a high removal rate of the SPTB layer after alkali texturing.

**Figure 5:**
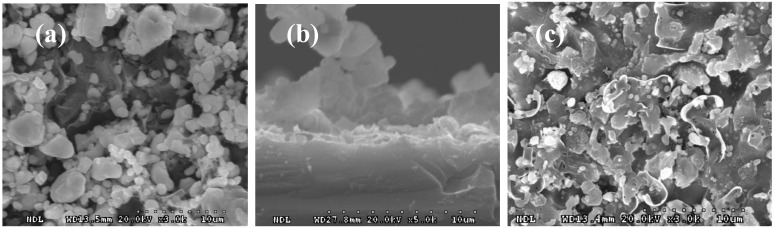

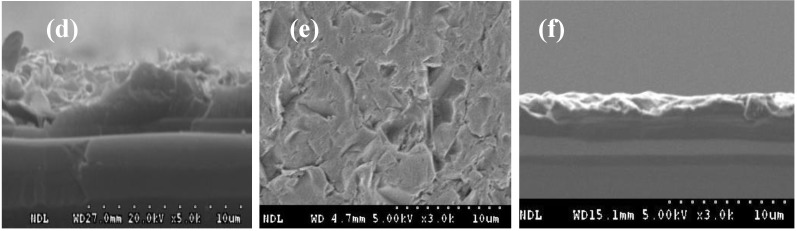
The SPTB layer/p-type Si(100) substrates were fired at 840 °C for 60 min and textured in 1.7% KOH solution for 25 min. Then, surface morphologies and cross-section images of the SPTB layer/p-type Si(100) substrates etched in mixed HCl/H_2_O_2_ solution at (**a**,**b**) 10, (**c**,**d**) 30, (**e**,**f**) 60 min, respectively, were detected by SEM.

The process sequences of ST are screen-printed SPTB paste, fired SPTB layer/p-type Si(100) substrate, texturing in KOH solution, and removal of the SPTB paste from the stacked SPTB layer/p-type Si(100) substrate. Thus, after alkali texturing of the SPTB layer/p-type Si(100) substrate, the SPTB layer should have been removed. To investigate the removal efficiency of the SPTB layer, the stacked SPTB layer/p-type Si(100) substrates were fired at 840 °C for 60 min and textured in 1.7% KOH solution for 25 min. Then, the surface morphologies and the cross-section images of the SPTB layer/p-type Si(100) substrate etched in the mixed HCl/H_2_O_2_ solution at (a,b) 10, (c,d) 30, (e,f) 60 min, respectively, were detected by SEM as shown in Figure 5. The results show that some SPTB residue was detected on the surface of the p-type Si(100) substrate etched in the mixed HCl/H_2_O_2_ solution at 10 min. Conversely, the SPTB layer can be removed under the SPTB layer/p-type Si(100) substrate etched in the mixed HCl/H_2_O_2_ solution at 60 min. The removal efficiency can be enhanced by increasing etching time.

**Figure 6:**
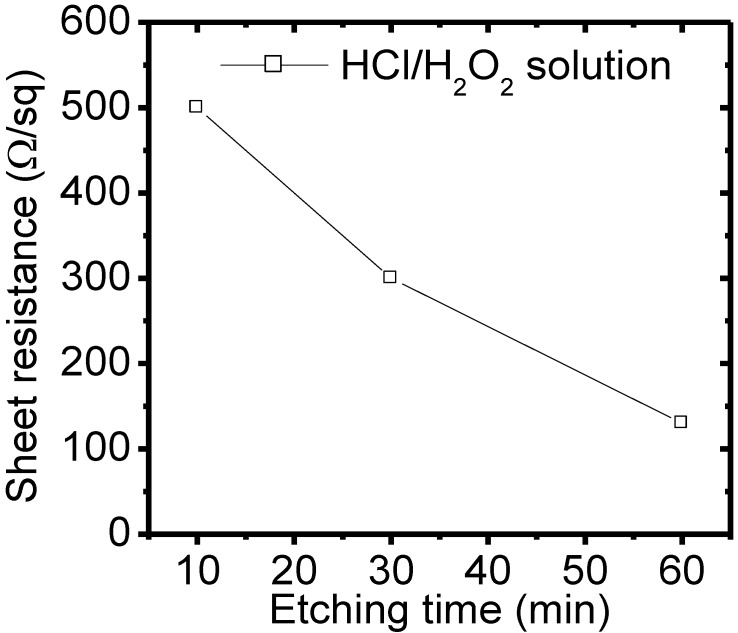
Sheet resistances of the SPTB layer/p-type Si(100) substrates etched in mixed HCl/H_2_O_2_ solution at 10, 30, and 60 min, respectively.

To confirm the SPTB residue on the p-type Si(100) surface, the sheet resistances of the p-type Si(100) surfaces with the removal of the SPTB layer were measured by four-point probe. The sheet resistances of the SPTB layer/p-type Si(100) substrate etched in mixed HCl/H_2_O_2_ solution at 10, 30, and 60 min, respectively, were measured and are shown in Figure 6. The sheet resistance of original p-type Si(100) substrate is around 130 Ω/sq. The removal efficiency is enhanced with increasing etching time. Compared with original sheet resistance, higher sheet resistance was obtained for the SPTB layer/p-type Si(100) substrate etched in mixed HCl/H_2_O_2_ solution at 10 min. This is due to the SPTB residue on the surface. On the other hand, the sheet resistance of the SPTB layer/p-type Si(100) substrate etched in mixed HCl/H_2_O_2_ solution at 60 min was around 130 Ω/sq. Thus, the SPTB layer was completely removed by the HCl/H_2_O_2_ solution at 60 min. These results were consistent with the results as shown in Figure 5.

**Figure 7:**
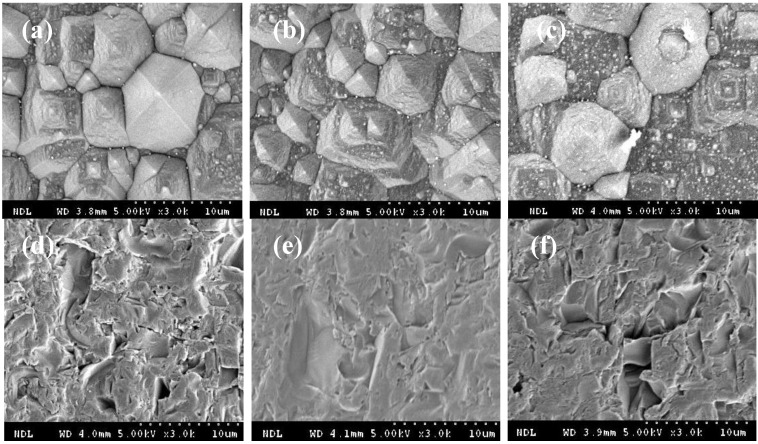
The SPTB layer/p-type Si(100) substrates were fired at (**a**) 10, (**b**) 20, (**c**) 40, (**d**) 60, (**e**) 80 and (**f**) 100 min, respectively. Then, all SPTB layer/p-type Si(100) substrates were textured in 1.7% KOH solution for 25 min. Finally, the SPTB layer/p-type Si(100) substrates were etched in mixed HCl/H_2_O_2_ solution for 60 min. All surface morphologies of the p-type Si(100) substrate after SPTB removal were detected by SEM.

Finally, to demonstrate the ST of the IBC silicon solar cell, the SPTB layer/p-type Si(100) substrates were fired at 10, 20, 40, 60, 80, and 100 min, respectively. Then, all SPTB layer/p-type Si(100) substrates were textured in 1.7% KOH solution for 25 min. Finally, all SPTB layer/p-type Si(100) substrates were etched in the mixed HCl/H_2_O_2_ solution for 60 min. Figure 7 shows all surface morphologies of the p-type Si(100) substrate after SPTB removal detected by SEM. Again, the slight texturing on the surface between the SPTB layer and p-type Si(100) substrate was obtained for the SPTB layer/p-type Si(100) substrate fired below 60 min. With short firing time of the SPTB paste, the KOH solution passes easily through the voids on the SPTB layer. Thus, the ST of the IBC silicon solar cell can be demonstrated by the SPTB layer/p-type Si(100) substrate fired above 60 min. These results are consistent with Figure 2.

## 4. Conclusions

The screen-printed texture-barrier (SPTB) paste for single-side texturization (ST) of IBC silicon solar cell applications has been demonstrated. The SPTB paste consists of silicate glass (75 wt %), a resin binder (20 wt %) and a dispersing agent (5 wt %). The results show that the voids in the SPTB layer were decreased with increased firing times for the SPTB layer. There is no texturization between the SPTB layer/p-type Si(100) substrates interface for the SPTB paste fired above 60 min. The SPTB layer can be removed by mixed HCl/H_2_O_2_ solution over 60 min. The complete removal of the SPTB layer can be demonstrated by the surface morphology and the sheet resistance of the silicon surface. Furthermore, the cost of the screen-printer and the high-temperature furnace is cheap, around half of that of plasma-enhanced chemical vapor deposition (PECVD). The cost of the SPTB is around $100/kg which is lower than the SiH_4_/NH_3_ gas ambient used in PECVD. Thus, the fabricated cost of the IBC solar cells can be reduced by this new texturization-barrier paste.
